# Interplay between Furin and Sialoglycans in Modulating Adeno-Associated Viral Cell Entry

**DOI:** 10.1128/jvi.00093-23

**Published:** 2023-04-25

**Authors:** Timothy J. Smith, Robert M. Fusco, Zachary C. Elmore, Aravind Asokan

**Affiliations:** a Department of Molecular Genetics & Microbiology, Duke University School of Medicine, Durham, North Carolina, USA; b Department of Biomedical Engineering, Duke University, Durham, North Carolina, USA; c Department of Surgery, Duke University School of Medicine, Durham, North Carolina, USA; Cornell University Baker Institute for Animal Health

**Keywords:** adeno-associated virus, cell surface, sialic acid, subcellular localization, virus-host interactions

## Abstract

Adeno-associated viruses (AAVs) are small, helper-dependent, single-stranded DNA viruses that exploit a broad spectrum of host factors for cell entry. During the course of infection, several AAV serotypes have been shown to transit through the *trans*-Golgi network within the host cell. In the current study, we investigated whether the Golgi-localized, calcium-dependent protease furin influences AAV transduction. While CRISPR/Cas9-mediated knockout (KO) of the *Furin* gene minimally affected the transduction efficiency of most recombinant AAV serotypes tested, we observed a striking increase in transgene expression (~2 log orders) for the African green monkey isolate AAV4. Interrogation of different steps in the infectious pathway revealed that AAV4 binding, uptake, and transcript levels are increased in furin KO cells, but postentry steps such as uncoating or nuclear entry remain unaffected. Recombinant furin does not cleave AAV4 capsid proteins nor alter cellular expression levels of essential factors such as AAVR or GPR108. Interestingly, fluorescent lectin screening revealed a marked increase in 2,3-*O*-linked sialoglycan staining on the surface and perinuclear space of furin KO cells. The essential nature of increased sialoglycan expression in furin KO cells in enhancing AAV4 transduction was further corroborated by (i) increased transduction by the closely related isolates AAVrh.32.33 and sea lion AAV and (ii) selective blockade or removal of cellular 2,3-*O*-linked sialoglycans by specific lectins or neuraminidase, respectively. Based on the overall findings, we postulate that furin likely plays a key role in regulating expression of cellular sialoglycans, which in turn can influence permissivity to AAVs and possibly other viruses.

**IMPORTANCE** Adeno-associated viruses (AAVs) are a proven recombinant vector platform for gene therapy and have demonstrated success in the clinic. Continuing to improve our knowledge of AAV-host cell interactions is critical for improving the safety and efficacy. The current study dissects the interplay between furin, a common intracellular protease, and certain cell surface sialoglycans that serve as viral attachment factors for cell entry. Based on the findings, we postulate that differential expression of furin in host cells and tissues is likely to influence gene expression by certain recombinant AAV serotypes.

## INTRODUCTION

Adeno-associated viruses (AAVs) are *Dependoparvoviruses* with wide-ranging tissue tropism and a natural lack of pathogenicity (1). The AAV genome contains two genes, *Rep* and *Cap*, encoding proteins essential for replication and capsid assembly, respectively, flanked by inverted terminal repeat hairpins, which are the only *cis*-acting elements required to generate recombinant vectors (2). Different AAV serotype capsids are known to engage a range of cell surface attachment factors and exploit different host factors for intracellular uptake and subsequent transduction (3). Specifically, AAV capsids can initiate attachment through an array of charged glycans such as heparan sulfate, *N*- and *O*-linked sialic acids, or galactosylated glycans in a serotype-dependent manner (4–9). Internalization and rapid retrograde transport to the Golgi and subsequent nuclear entry through endocytic pathways are mediated by the essential pan-serotype receptor AAVR (KIAA0319L) (10, 11). The Golgi-localized G protein-coupled receptor (GPR108) has also been recently identified as an essential host factor for AAV transduction, although the specific postentry steps are less defined (12, 13). Certain exceptions have been noted, in particular, AAV4 and related isolates, which is known to be AAVR-independent but requires GPR108, while AAV5 is thought to be GPR108-independent but requires AAVR for infection (12, 14). Regardless of these differences, numerous studies implicate the Golgi apparatus as a critical intracellular compartment preceding AAV nuclear import, although the precise role of this step remains unclear (15–17).

Previous studies by our lab and others have revealed the Golgi calcium ATPase pump (SPCA1) as an essential host factor for AAVs and other viruses within the *Paramyxoviridae*, *Flaviviridae*, and *Togaviridae* families (18, 19). Specifically, we demonstrated that knockout of SPCA1 did not affect AAV binding, uptake, or nuclear entry but blocks AAV trafficking through the *trans*-Golgi network. Notably, while AAV was still able to enter the nucleus, the transcriptional efficiency of AAV capsid-associated vector genomes decreased considerably. These earlier findings support the notion that intracellular gradients and viral processing within the Golgi are critical for AAV transduction. Relatedly, a prominently studied Golgi protein that is markedly affected by calcium gradients is the ubiquitous serine endoprotease furin (19). Numerous mammalian protein substrates, including growth factors, cytokines, hormones, enzymes, and receptors, require proteolytic activation by furin and have been summarized previously (20). Furthermore, several viruses exploit the proteolytic processing of furin for viral entry (for instance, the minor capsid protein L2 of human papillomavirus and premembrane protein of Dengue virus) and for maturation and egress (Env of HIV and spike protein of coronavirus) (21–24).

In this study, we explored the role of furin in the AAV infectious pathway. Furin knockout (KO) cells were generated to assess transduction of a panel of AAVs. We found that serotype AAV4 had a substantial increase in transduction efficiency in the absence of furin. We then systematically evaluated how furin might play a role in AAV biology. First, we found that furin did not cleave AAV4 capsid protein subunits *in vitro* or appreciably effect AAV host factors AAVR or GPR108. We then characterized virus-host cell interactions and found a significant increase in AAV4 binding and cellular uptake in cells lacking furin. We showed that this gain-of-function phenotype is caused by an increase in *O*-linked sialoglycans in the absence of furin and extends to other AAV4-like serotypes. This work highlights a novel interplay between furin and sialylated glycoproteins, which in turn can influence the permissivity of host cells for AAV transduction.

## RESULTS

### Furin is a restrictive host factor for adeno-associated virus 4 transduction.

**FIG 1:**
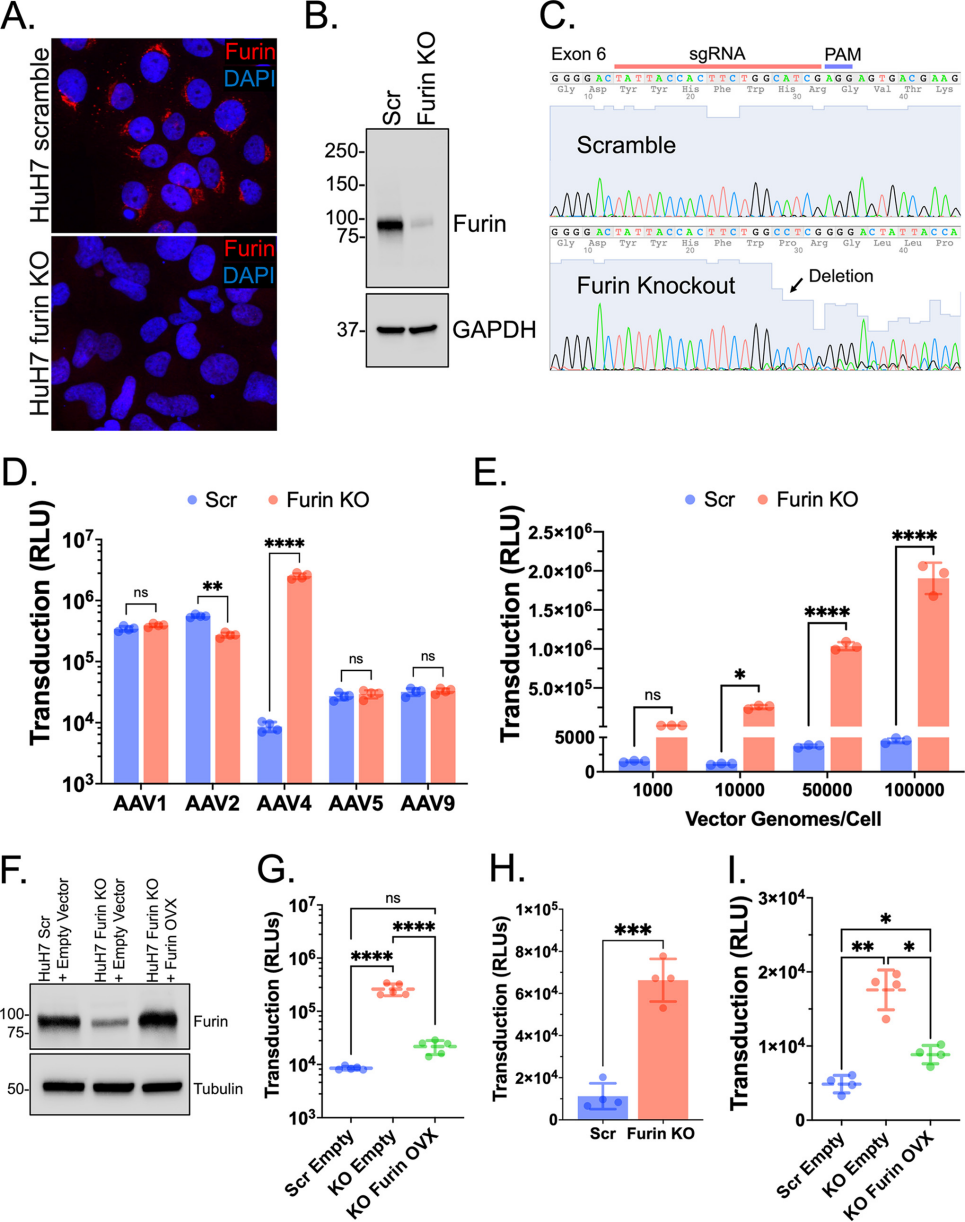
Furin knockout (KO) increases AAV4 transduction. (A) Immunofluorescent staining of furin in HuH7 scramble (scr) and furin KO cell lines. (B) Western blot analyses of furin protein expression in HuH7 scramble and furin KO cells. (C) Sanger sequencing showing disruption of the *furin KO* target region. (D) Transduction of HuH7 scramble and furin KO cell lines with various adeno-associated virus (AAV) serotypes. (E) Transduction of AAV4 across multiple multiplicities of infection (MOIs). (F) Western blot of HuH7 whole-cell lysate following transient transfection of empty vector or pcDNA3.1-furin. (G) AAV4 transduction of HuH7 cell lines transiently transfected with empty vector or pcDNA3.1-furin. (H) AAV4 transduction in HEK 293 scramble or furin KO cells. (I) AAV4 transduction of HEK 293 cells transiently transfected with empty vector or pcDNA3.1-furin. All serotypes were transduced at MOI of 50,000 vg/cell except for AAV2, which was transduced at 1,000 vector genomes/cell. Error bars denote means ± standard deviation. Statistical significance was determined using two-way analysis of variance (ANOVA) (transduction assays) and one-way ANOVA (overexpression assays) with Šidák’s correction for multiple comparisons. Analyses were performed with GraphPad Prism version 9 with *P* values less than 0.05 being significant. *, *P* < 0.05; **, *P* < 0.01; ***, *P* < 0.001; ****, *P* < 0.0001. sgRNA, single guide RNA; OVX, overexpression; PAM, protospacer-adjacent motif.

To investigate the role of furin in AAV4 transduction, we created isogenic knockout cell lines in human hepatocarcinoma (HuH7) cells using CRISPR-Cas9 genome editing. Briefly, guide RNAs targeting an exonic region upstream of the active site were ligated into LentiCRISPRv2 vector (25), packaged into recombinant lentivirus, and used to transduce HuH7 cells. Scramble control cells were generated in parallel using nontargeting control guides. Antibiotic selection and serial dilutions were used to isolate a clonal furin KO population. Antibodies were unable to detect furin protein by immunofluorescent staining (Fig. 1A), and Western blotting showed substantial knockdown of furin protein expression in furin KO cells (Fig. 1B). Sanger sequencing indicated mutation within the single guide RNA (sgRNA) target region, further confirming furin KO (Fig. 1C).

Following validation of cell lines, HuH7 scramble control and furin KO cells were transduced with AAV1, AAV2, AAV4, AAV5, and AAV9 packaging a luciferase reporter transgene driven by a chicken β-actin promoter. While most AAV serotypes were unaffected, transduction for AAV4 markedly increased over 200-fold compared to scramble control cells (Fig. 1D). We tested this across various multiplicities of infection (MOI) ranging from 1,000 to 100,000 vector genomes per cell (vg/cell), and indeed, we observed a trend of increasing transduction efficiency for AAV4 (Fig. 1E). Next, we wanted to see whether transient overexpression of furin KO cells could rescue the wild-type phenotype. Empty pcDNA3.1 or pcDNA3.1-furin were transiently transfected into HuH7 cell lines, and expression of furin protein in knockout cells was confirmed by Western blotting (Fig. 1F). Furin KO cells transfected with empty vector showed increased transduction compared to scramble (Fig. 1G). In contrast, furin KO cells transfected with pcDNA3.1-furin showed a decrease in transduction to nearly wild-type levels (Fig. 1G). AAV4 transduction and overexpression experiments were also conducted in HEK 293 cell lines bearing furin KO and confirmed by Western blotting (data not shown). AAV4 transduction increased 6-fold in the absence of furin and transient furin overexpression in furin KO cells partially rescued the wild-type phenotype (Fig. 1H and I). These results implicate furin as an inhibitory host factor on AAV4 transduction.

### Furin does not proteolytically cleave AAV4 capsid protein subunits.

**FIG 2:**
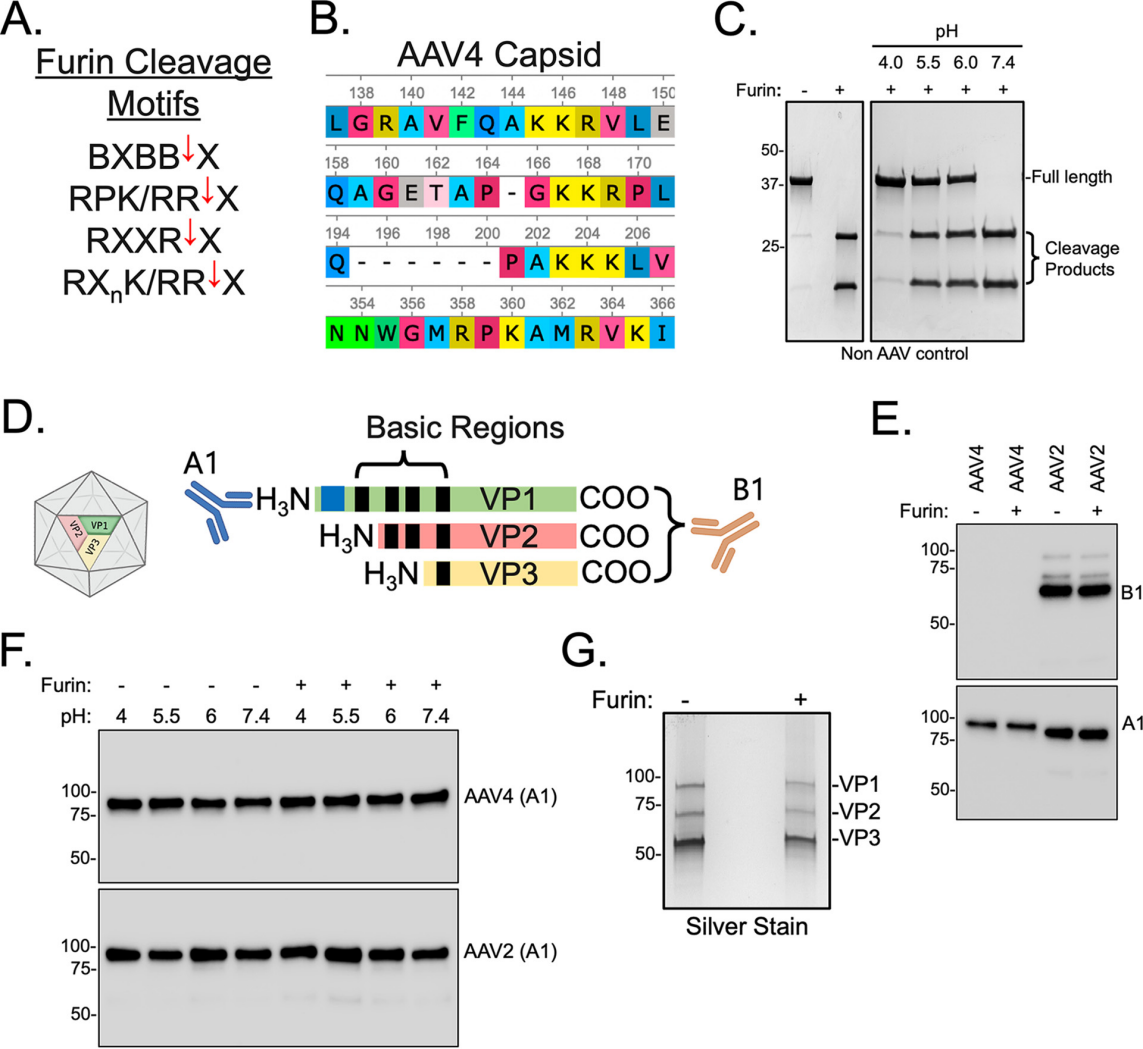
Furin does not affect AAV4 capsid protein integrity or expression of cellular factors AAVR and GPR108. (A) General furin protease recognition motif listed with common examples in which B is a basic amino acid, and X is a hydrophobic amino acid with the red arrow indicating a cleavage site. (B) Basic amino acid footprints located within the AAV4 capsid sequence. (C) Western blot of control substrate MBP5-paramyosin-ΔSal following treatment with vehicle or recombinant human furin enzyme at various pH. Its predicted molecular weight is 69 kDa, and after successful hydrolysis by furin, it produces two fragments of 42 and 27 kDa. (D) Schematic detailing AAV capsid protein subunits VP1 (87 kDa), VP2 (72 kDa), and VP3 (62 kDa). A1 monoclonal antibody binds to an N-terminal region present only on the VP1 capsid subunit, whereas B1 recognizes an epitope on the C termini of VP1, 2, and 3. (E, F) Western blot for A1 or B1 of purified AAV4 virions treated with vehicle or recombinant furin enzyme at various pH. (G) silver staining of an SDS-PAGE gel with AAV4 capsid untreated or treated with recombinant furin enzyme.

Given the inhibitory effect furin exerts on AAV4, we sought to investigate whether the capsid protein subunits are targets for furin proteolytic activity. Furin prefers basic amino acid residues to hydrolyze proteins, and while the canonical motif is Arg-X-Arg/Lys-Arg-X with cleavage occurring between the last arginine and the hydrophobic X residue, numerous variations (Fig. 2A) of the cleavage site have been documented and can span up to 20 residues from the active site (26). In corollary, there are multiple clusters of basic amino acid residues within the AAV capsid that could be a target for furin cleavage (Fig. 2B) (27, 28). To test whether furin acts on the capsid protein subunits, we treated purified AAV4 virions with recombinant human furin enzyme under various pH to mimic cytoplasmic (pH 7.4), early (pH 6.0), and late (pH 5.5) endosomal conditions. MBP5-paramyosin-ΔSal is a maltose-binding fusion protein (MBP) containing a furin cleavage site between the MBP and paramyosin domains and was used as a positive control to confirm furin enzymatic activity across different pH by Western blotting (Fig. 2C). We then utilized the mouse monoclonal antibodies B1, directed at an epitope on the C terminus of VP1/VP2/VP3, and A1, directed to an epitope on N terminus of VP1 capsid protein to assess cleavage by furin (Fig. 2D). We observed no cleavage of AAV4 or negative-control AAV2, capsid subunits (Fig. 2E) or across different pH levels (Fig. 2F) as shown by Western blotting. As AAV4 capsid protein subunits cannot be recognized by the B1 antibody, we assessed cleavage of all three subunits (VP1, 2, and 3) by silver staining of a native gel and confirmed that no cleavage was observed (Fig. 2G).

### The AAV4 host factor GPR108 shows moderately altered cellular localization upon furin KO.

**FIG 3:**
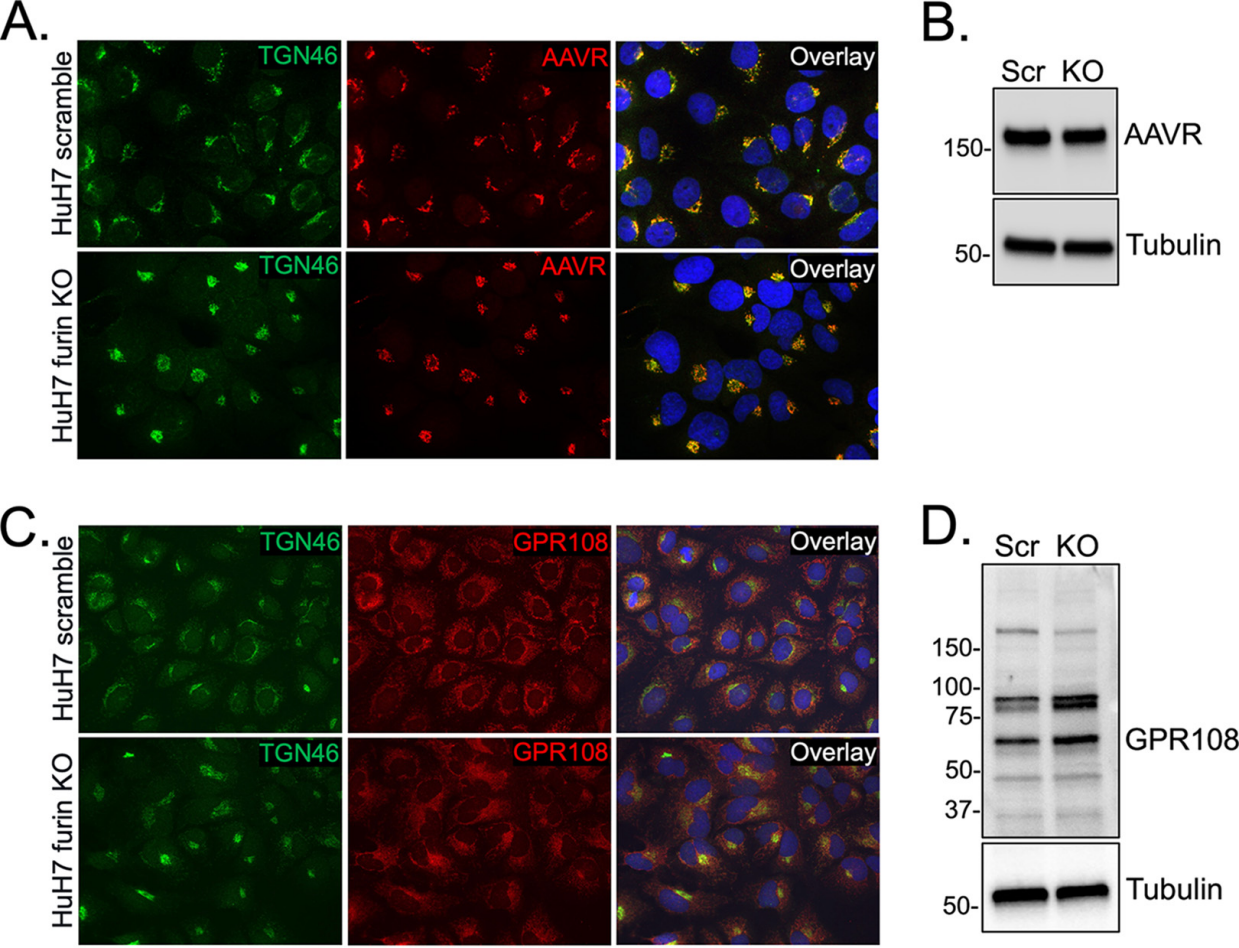
Furin does not significantly alter expression of cellular factors AAV receptor (AAVR) and GPR108. (A, C) Immunofluorescent staining of endogenous AAVR or GPR108 (red) with trans-Golgi network marker TGN46 (green) and 4′,6-diamidino-2-phenylindole (DAPI) (blue) in HuH7 scramble or furin KO cells. (B, D) Western blot of normalized whole-cell lysate for endogenous AAVR, GPR108, or α-tubulin loading control.

Our next step was to evaluate whether furin KO affects AAV host factors in a way that also affects AAV4 trafficking. Two essential host factors, AAVR and GPR108, have been reported for AAV transduction (11, 12). Although AAV4 is thought to be AAVR-independent and GPR108-dependent (14), we assessed the expression and cellular localization of both membrane proteins in furin KO and scramble control cells. Immunofluorescent staining with AAVR and a marker for the *trans*-Golgi network (TGN46) indicated that the cellular expression levels and localization of AAVR were unaffected by furin KO (Fig. 3A). AAVR expression was also probed by Western blotting of normalized whole-cell lysate. Likewise, no difference was observed in the expression pattern between scramble and furin KO cells (Fig. 3B). Immunofluorescent staining of GRP108 and TGN46 showed a slightly altered localization pattern (Fig. 3C), while Western blotting for GPR108 showed only a modest increase in GPR108 protein expression in furin KO cells compared to scramble (Fig. 3D). Although interesting, we conclude that these modest changes are unlikely to account for the significantly improved transgene expression profile observed for AAV4.

### Furin KO significantly increases AAV4 binding and uptake, while the nuclear-cytoplasmic ratio remains relatively unaffected.

**FIG 4:**
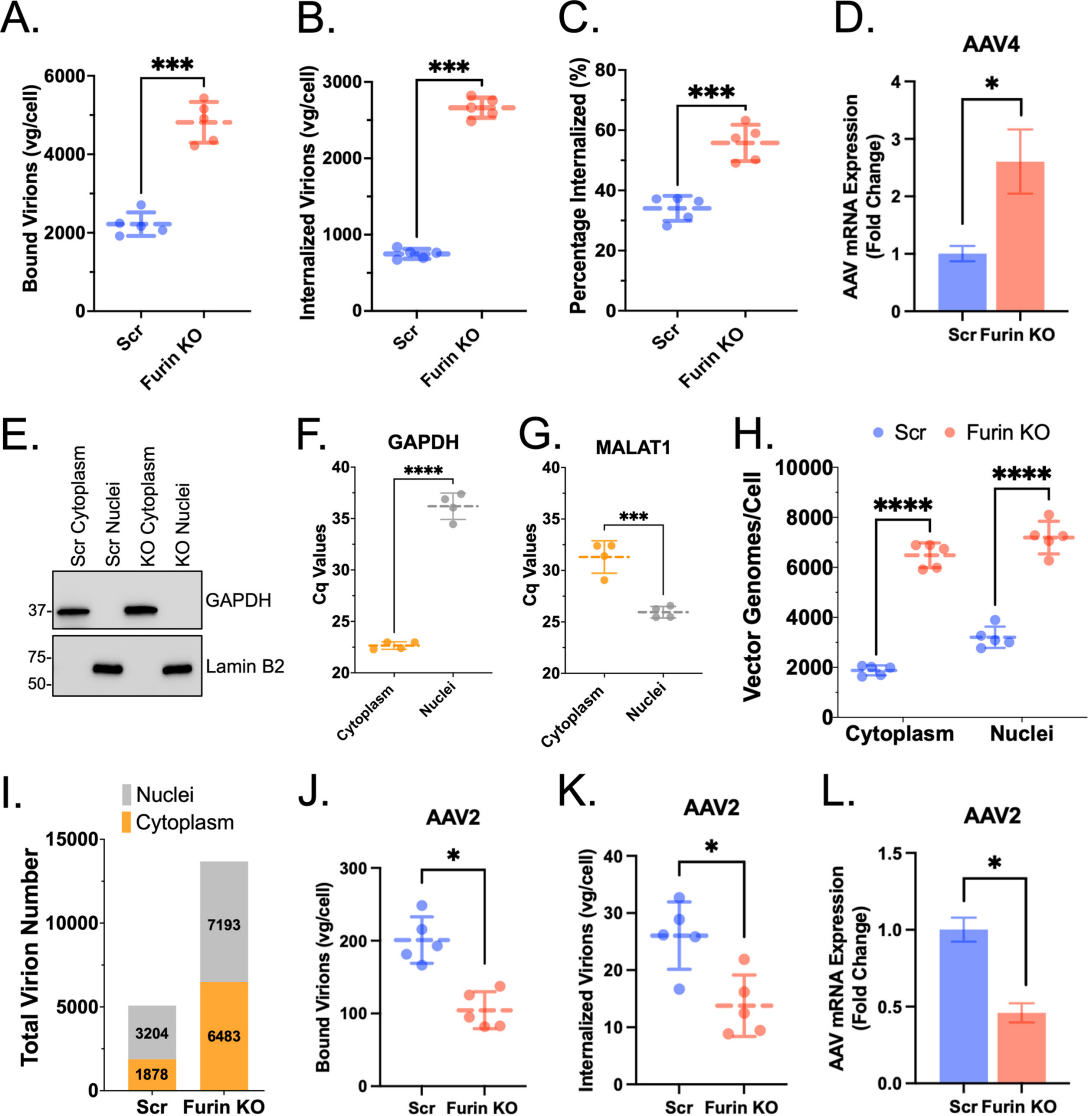
Furin KO increases AAV4 cellular binding and internalization. (A, B) Total number of bound (A) or internalized (B) AAV4 virions per cell isolated from HuH7 scramble or furin KO cells quantified by quantitative PCR (qPCR). (C) Percentage of AAV4 virions internalized. (D) Relative levels of mRNA transcribed from AAV4 vector genomes. (E) Western blot of HuH7 scramble or furin KO subcellular extracts for cytoplasmic (glyceraldehyde-3-phosphate dehydrogenase [GAPDH]) and nuclear (LaminB2) markers. (F, G) Quantitative PCR analysis of cytoplasmic (GAPDH) or nuclear (MALAT1) gene targets. (H) Total number of vector genomes per cell isolated from cytoplasmic or nuclear fractions 16 h post-transduction with AAV4 quantified by qPCR. (I) Total virion number localized to each subcellular compartment quantified by qPCR. (J to L) Binding (J), uptake (K), and mRNA transcript levels (L) for control serotype AAV2. The cells were transduced at 50,000 vg/cell. Error bars denote mean ± standard deviation. For binding and uptake assays, statistical significance was determined using a two-tailed unpaired *t* test with Welch’s correction. For cytoplasmic/nuclear fraction assays, statistical significance was determined using a two-way ANOVA with Šidák’s correction for multiple comparisons. mRNA expression fold change was determined with the ΔΔ*CT* method, and the data were log transformed for statistical analysis using two-tailed unpaired *t* test. Analyses were performed using GraphPad Prism version 9 with *P* values less than 0.05 being significant. *, *P* < 0.05; **, *P* < 0.01; ***, *P* < 0.001; ****, *P* < 0.0001. vg, vector genomes.

Next, we performed viral binding and uptake assays described previously (29). Briefly, we incubated AAV4 virions with HuH7 scramble or furin KO cells at 4°C followed by removal of unbound virions. For uptake, bound virions were subject to cellular internalization at 37°C and vector genomes per cell determined by qPCR. We observed a significant (2.2-fold) increase in the number of virions bound to the surface of furin KO cells compared to scramble (Fig. 4A). Correspondingly, there was also a 3.6-fold increase in cellular uptake (Fig. 4B) and the percentage of virions internalized increased from 34% to 55% in furin KO cells (Fig. 4C). We also observed a statistically significant (2.6-fold) increase in transcribed mRNA in furin KO cells compared to scramble consistent with the overall increase in binding and entry. (Fig. 4D). We then investigated whether the absence of furin affects nuclear localization by transducing cells with AAV4 for 18 h and then separating cytoplasmic and nuclear extracts. Fractionation was confirmed by Western blotting for glyceraldehyde-3-phosphate dehydrogenase (GAPDH) and LaminB2 for cytoplasmic and nuclear fractions, respectively (Fig. 4E). To further confirm cytoplasmic and nuclear separation, we performed qPCR to compare the *C_q_* values for target genes GAPDH (Fig. 4F) and MALAT1 (Fig. 4G) between different fractions. We then determined vector genome numbers by qPCR. While there was an increase in both cytoplasmic and nuclear localized virions in furin KO cells, the cytoplasmic-to-nuclear ratio remained relatively similar in scramble and furin KO cell lines (Fig. 4H and I). Lastly, we tested a different serotype, AAV2, which utilizes heparan sulfate for cell surface attachment to assess binding, entry, and mRNA transcript levels. We observed lower binding, uptake, and mRNA levels that are consistent with the observed decrease in transduction observed earlier (Fig. 4J to L). Taken together, these data support an increase in the ability of AAV4 virions to bind and enter the cell as the likely event leading to enhanced transduction in the absence of furin.

### Furin knockout enhances transduction of other AAV4-like serotypes.

**FIG 5:**
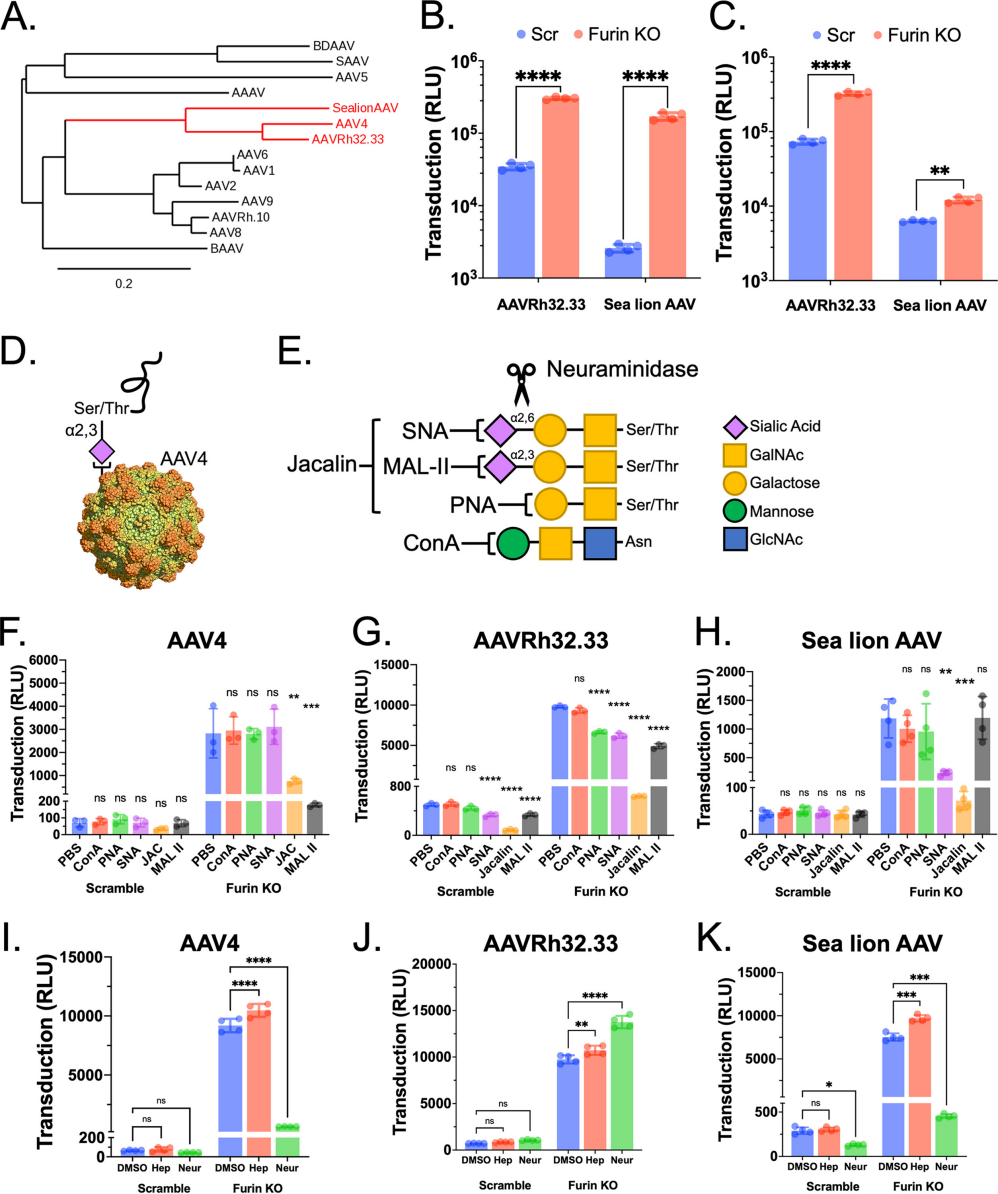
Furin KO enhances transduction of other AAV4-like serotypes in a sialic acid-dependent manner. (A) Evolutionary lineage of a subset of AAV serotypes and isolates with AAV4-like serotypes highlighted in red. Branch length is proportional to the amount of inferred evolutionary change and the distance scale for branch length is noted on the bottom left. SAAV, serpentine AAV; BAAV, bat AAV; BDAAV, bearded dragon AAV. (B, C) Transduction of HuH7 (B) or HEK 293 (C) scramble and furin KO cell lines with AAVrh32.22 or sea lion AAV. (D) AAV4 uses *O*-linked α2,3-sialic acid for cellular binding. (E) Overview of lectin binding preference for Concanavalin A (ConA; *N*-linked glycans), Sambucus nigra lectin (SNA; α2,6-sialic acid), Peanut Agglutinin (PNA; nonsialylated T antigen), Jacalin (JAC; *O*-linked glycans), Maackia amurensis lectin II (MAL II; α2,3-sialic acid). (E) Neuraminidase (neur) was used for enzymatic removal of sialic acid from the cell surface. (F to K) Cells were pretreated with lectins (F to H), neuraminidase, or heparinase (hep) (I to K) prior to the addition of AAV, and transduction was assessed by measuring luciferase transgene expression. All cells were transduced with AAV4, AAVrh32.33, or sea lion AAV at 50,000 vg/cell. Error bars denote mean ± standard deviation. For transduction assays, statistical significance was determined using a two-way ANOVA. For lectin and enzymatic assays, statistical significance was determined using an ordinary one-way ANOVA with Šidák’s correction for multiple comparisons. Analyses were performed with GraphPad Prism version 9 with *P* values less than 0.05 being significant. *, *P* < 0.05; **, *P* < 0.01; ***, *P* < 0.001; ****, *P* < 0.0001. DMSO, dimethyl sulfoxide; PBS, phosphate-buffered saline.

To date, numerous human and primate AAV serotypes and genomic isolates have been identified with VP1 amino acid sequence identity across AAVs varying from 55% to 99% (30). We sought to investigate whether the enhanced transduction observed for AAV4 in furin KO cells can be recapitulated by isolates with similar lineage to AAV4, in particular, AAVrh32.33 and California sea lion AAV (Fig. 5A). Briefly, HuH7 scramble control and furin KO cells were transduced with AAVrh32.33 and sea lion AAV packaging a luciferase reporter transgene driven by a chicken β-actin promoter. Transduction for AAVrh32.33 and sea lion AAV increased over 8-fold and 52-fold, respectively, compared to scramble control cells (Fig. 5B). Similar to AAV4, we observed a lesser yet significant increase in transduction for AAVrh32.33 (4-fold) and sea lion AAV (2-fold) in HEK 293 cell lines (Fig. 5C). These data suggested that the inhibitory effect of furin on AAV transduction may be mediated through a host factor common to AAV4-like serotypes.

### Blocking sialic acid decreases AAV4 transduction in furin KO cells.

Our lab and others have demonstrated that cellular attachment *in vitro* and tissue tropism of AAV4 *in vivo* is dependent on expression of *O*-linked α2,3-sialylated glycans (5, 9) (Fig. 5D). To probe whether these interactions are altered in furin KO cells, we utilized a panel of lectins specific for *N*-linked glycans (ConA), *O*-linked glycans (Jacalin), nonsialylated T-antigen (PNA), sialylated α2,6-glycans (SNA), and sialylated α2,3-glycans (MAL II) to competitively inhibit AAV binding (Fig. 5E). Scramble and furin KO cells were preincubated with phosphate-buffered saline (PBS) or lectins and then transduced with AAV4. Notably, lectins that bind *O*-linked glycans and α2,3-sialylated glycans completely abrogate the increased transduction observed in furin KO cells, whereas lectins that bind other glycan linkages did not affect transduction (Fig. 5F). Similarly, pretreatment of cells with neuraminidase to remove surface sialic acids completely abolished the marked increase in AAV4 transduction observed in furin KO cells, whereas the removal of glycosaminoglycans using heparinase did not affect AAV4 transduction (Fig. 5I). Consistently, transduction by both AAVrh32.33 and sea lion AAV was also blocked by lectins that bind *O*-linked glycans (Fig. 5G and H). However, transduction by AAVrh32.33 was also partially blocked by other lectins, and interestingly, pretreatment of cells with neuraminidase increased transduction (Fig. 5G and J). These data suggest that AAVrh32.33 may utilize additional mechanisms for cellular attachment. Transduction by sea lion AAV was blocked by lectins specific for α2,6-sialylated glycans, and pretreatment of cells with neuraminidase also abrogated sea lion AAV transduction in furin KO cells (Fig. 5H and K). Despite these subtle differences in sialic acid linkage preference, the overall data corroborate the notion that furin KO cells display alterations in sialoglycan expression, which can render cells more permissive to AAV4, AAVrh32.33, and sea lion AAV.

### Furin KO increases the abundance and perinuclear localization of sialoglycans.

**FIG 6:**
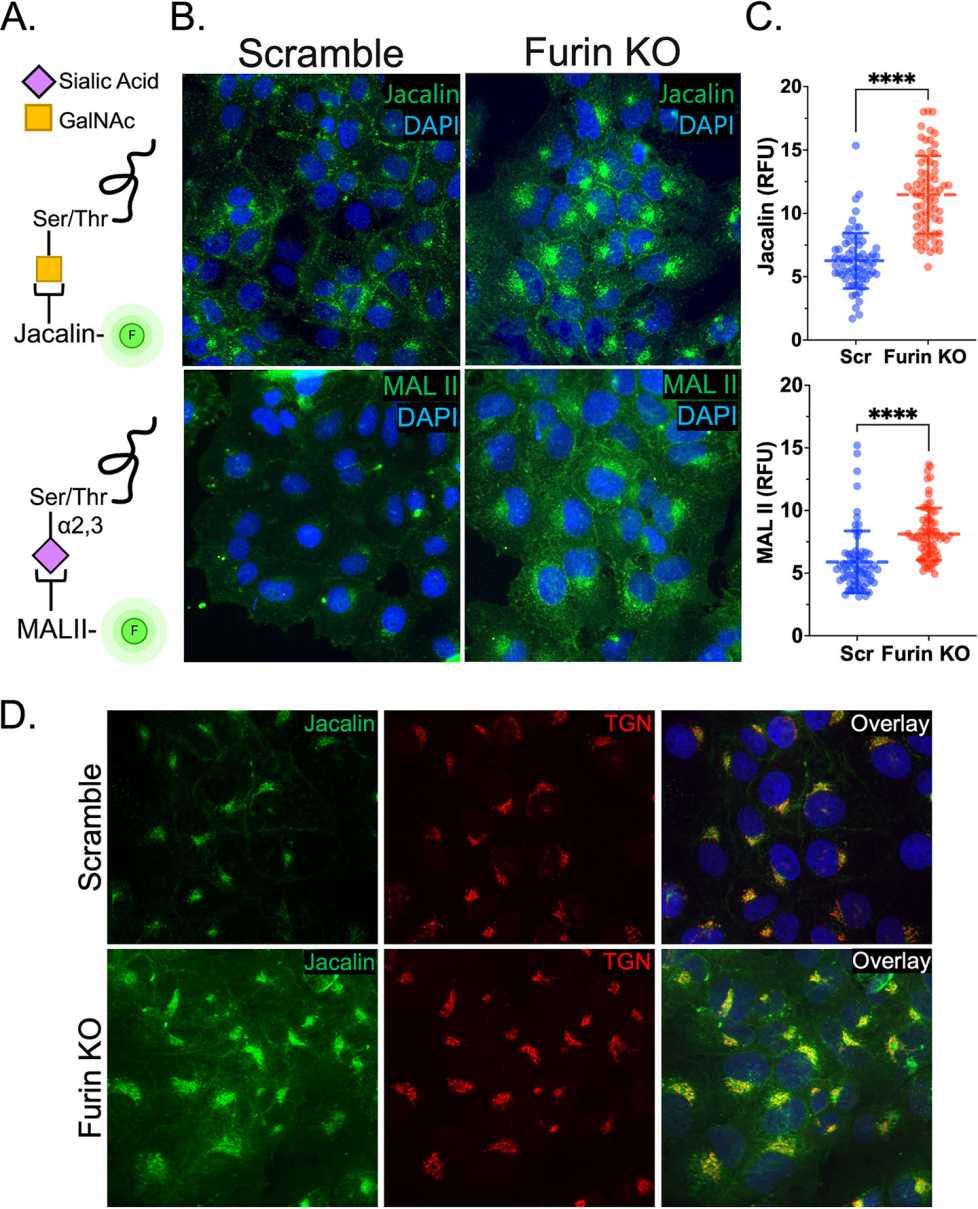
Furin KO increases the abundance and perinuclear localization of sialoglycans. (A) Endogenous expression of *O*-linked sialic acid was assessed in HuH7 scramble or furin KO cells using fluorescently conjugated lectins. (B) Immunofluorescent staining for *O*-linked glycosylation was carried out using jacalin (top panels) and for α2,3-sialic acid using MAL II (bottom panels). (C) Fluorescence in panel B was quantitated by counting 70 cells from multiple images and analyzed for relative fluorescent units using ImageJ. (D) Immunofluorescent staining for lectins and trans-Golgi network marker TGN46. Error bars denote mean ± standard deviation. To determine statistical significance, a two-tailed unpaired *t* test was performed using GraphPad Prism version 9 with *P* values less than 0.05 being significant. *, *P* < 0.05; **, *P* < 0.01; ***, *P* < 0.001; ****, *P* < 0.0001.

**FIG 7:**
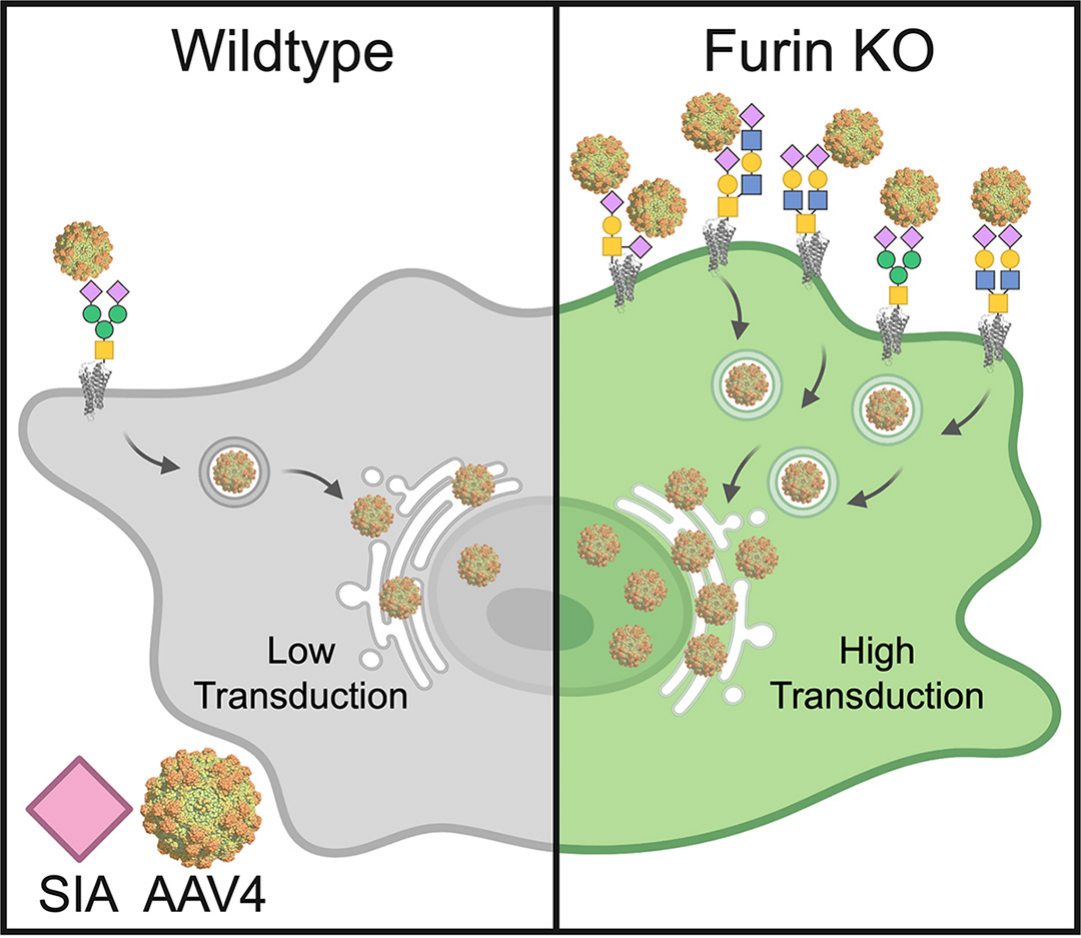
Intracellular model delineating the potential role of furin interplay with sialoglycans in AAV transduction. Furin is a *trans*-Golgi-localized host factor that restricts AAV4, and related serotypes, transduction in HuH7 and HEK 293 cells lines. In the absence of furin, AAV4 displays enhanced binding and cellular uptake. This gain-of-function phenotype is caused by an increase in the amount of *O*-linked sialylated glycans to which these AAVs can bind to on the cell surface. By a yet to be determined mechanism, furin regulates the abundance of *O*-linked sialic acid. Schematics were created using Biorender.com.

To further establish a link between furin and sialoglycoprotein expression, HuH7 scramble and furin KO cells were stained using the fluorescently conjugated lectins Jacalin and MAL II (Fig. 6A). We observed a substantial increase in the amount of *O*-linked α2,3-sialoglycans in furin KO cells compared to scramble (Fig. 6B and C, top). Concomitantly, there was an increase in the perinuclear localization of *O*-linked α2,3-sialoglycans (Fig. 6B and C, bottom). Additionally, we observed significant colocalization of Jacalin with the *trans*-Golgi network marker TGN46 (Fig. 6D). This supports the notion that enhanced binding and uptake leads to increased trafficking of virions to the perinuclear space, and ultimately, increased transduction. Overall, these data demonstrate that the marked increase in AAV4 transduction in the absence of furin is modulated by increased levels of sialoglycoproteins (Fig. 7).

## DISCUSSION

Furin is an essential proprotein convertase belonging to the subtilisin superfamily of serine endoproteases (31). While broadly expressed across tissues, the level of furin expression varies considerably from tissue to tissue, with higher levels in the kidney and brain and lower levels in the heart and lung. At the subcellular level, autocatalytic events in the endoplasmic reticulum (ER), as well as the TGN, are essential to produce the catalytically active version of the enzyme. The enzyme then follows a tightly regulated trafficking pattern between the ER, the TGN, and the cell surface, lending to its ability to cleave a multitude of cellular prosubstrates (20). Concomitant with localization in ER/TGN, furin is strictly calcium-dependent and has a high propensity to bind and cleave protein substrates at clusters of basic Arg/Lys residues as indicated earlier. Furin has been implicated in the processing of a number of viral proteins required for cellular entry and infection. For instance, furin is responsible for cleavage of the HIV-1 surface protein gp160 into gp120 and gp41, hemagglutinin of multiple avian influenza viruses, S protein of Middle Eastern respiratory syndrome coronavirus, and membrane protein cleavage of various flaviviruses including dengue, West Nile, and Zika virus (23, 31–35). More recently, furin has been shown to play a critical role in cleaving the S protein of severe acute respiratory syndrome coronavirus 2 (SARS-CoV-2) to help mediate cell-cell and virus-cell fusion *in vitro* (36). The current study further expands our understanding of the role played by furin in viral infection by discovering that host transduction by certain AAV strains, particularly those utilizing *O*-linked sialoglycans for cellular attachment, are influenced by such mechanisms. Strikingly, while most studies to date demonstrate that specific viral proteins are furin substrates, AAV capsid proteins do not appear to be degraded *in vitro* by furin, despite possessing several basic clusters of amino acid residues in the VP1 capsid protein subunit. In contrast, we observe that a broad spectrum of host sialoglycoproteins are markedly upregulated and took on a more perinuclear localization pattern upon genetic ablation of furin from host cells. This phenomenon, in turn, appears to help increase the transduction efficiency of AAV4 and other related viral strains.

Another interesting observation in the current study is that the expression levels of two notable membrane proteins, GPR108 and AAVR, are unaffected by furin KO. While AAV4 and AAVrh32.33 are known to be independent of AAVR, both isolates require GPR108. A closer examination of immunofluorescent micrographs detecting GPR108 revealed changes in intracellular trafficking patterns of this protein in HuH7 furin KO cells compared to scramble control. Whether such subcellular reordering/mislocalization of GPR108 favorably affects AAV4 trafficking and consequently, improved transduction requires further investigation. Given the established importance of trafficking to the TGN for AAV transduction, it is conceivable that increased binding to sialylated glycans coupled with increased trafficking, perhaps with GPR108, to the TGN enables more transduction to occur. Moreover, the glycosylation profile (sialylation, in particular) of GPR108 is currently unknown and may shed more light on the intracellular trafficking mechanisms hijacked by AAV4 capsids. Nevertheless, our combined findings pertaining to (i) an earlier report from our lab regarding the role of Golgi calcium levels (as modulated by the calcium ATPase pump [SPCA1]) and (ii) the current report highlighting the role of furin herein further corroborate the critical role of AAV capsid trafficking through the TGN for productive infection.

Another key aspect of the current study is the continued emphasis on the unique biology of the divergent serotypes, AAV4 and AAVrh32.33, compared to other isolates. Previous reports by our lab and others have demonstrated a range of unique attributes displayed by AAV4 and/or AAV.rh32.33, notably, unique 3D capsid structural features, capsid assembly independent of accessory proteins (AAP), the exploitation of distinct cell surface sialoglycans for attachment, AAVR independence, requirement of GPR108 for infection, cardiopulmonary tropism in mice, unique CNS transduction profile, low seroprevalence, and the unique nature of host cellular immune responses to these capsids (5, 9, 12, 14, 28, 37–44). We expand on these findings by demonstrating that cellular furin levels can modulate cellular expression levels of sialoglycans, which in turn affects AAV4 and AAVrh32.33. In addition, we add a new member to this family of divergent AAV isolates: California sea lion AAV, which appears to be affected similarly by the interplay between furin and sialoglycan levels.

The exact mechanism(s) by which furin KO results in upregulation of sialoglycans (or sialoglycoproteins thereof) remain to be determined. Furin is generally known to proteolytically process a spectrum of mammalian targets including extracellular matrix proteins, integrins, signaling peptides, hormones, growth factors, and many serum proteins, such as albumin, coagulation factors, complement proteins, and a number of membrane-bound receptors (20). Therefore, one explanation is that the observed furin KO-mediated increase in sialoglycoproteins is largely part of a more pleotropic effect. However, this is patently not the case, because we do not observe serotype-independent increase in AAV transduction. In fact, only serotypes utilizing 2,3-*O*-linked sialoglycans appear to be affected. Additional RNA-sequencing (RNA-seq) assessment of different furin KO cell lines (outside the scope of the current study) may shed light on such mechanisms and provide insights that are broadly applicable in the context of viral infection. Lastly, the impact of furin on the tissue tropisms of different AAVs, particularly the unique isolates highlighted in the current study *in vivo* in different animal models, remains to be investigated further. When evaluating a subcellular map of the human proteome within the Human Protein Atlas, furin protein expression levels vary in a cell-type-dependent manner with little or no furin detected in the heart and lung. While this is only speculative, this expression pattern is in line with the *in vivo* cardiopulmonary tropism of AAV4 observed by our lab previously (9). Furin KO is known to be embryonic lethal in murine models, although inducible systems and small molecule inhibitors may allow interrogation of furin-mediated regulation of the host proteome and the potential impact of such on AAV tissue tropism *in vivo* (45). Such future studies may also enable the development of new strategies to improve gene transfer efficiency of recombinant AAV vectors by manipulating furin-host interplay.

## MATERIALS AND METHODS

### Tissue culture.

Human hepatocarcinoma (HuH7) and human embryonic kidney (HEK 293) cells lines were maintained at 37°C and 5% CO_2_ in Dulbecco’s modified Eagle’s minimal medium (DMEM; Gibco 11995073) supplemented with 10% fetal bovine serum (FBS; Sigma F0926) and 100 U/mL penicillin and 100 μg/mL streptomycin (Gibco 1514014) according to instructions from the American Type Culture Collection.

### Furin KO cell line generation.

CRISPR-Cas9 genome engineering was used to generate isogenic *furin* knockout in HuH7 and HEK 293 cell lines. Oligonucleotide guide RNA targeting exon 6 within the *furin* gene were designed using CHOPCHOP (https://chopchop.cbu.uib.no) and ordered from Integrated DNA Technologies (5′-TATTACCACTTCTGGCATCG-3′) (46). A nontargeting, scramble sequence was used as a negative control (5′-GCACTACCAGAGCTAACTCA-3′). Guides were phosphorylated, annealed, and ligated into a LentiCRISPRv2 plasmid (lentiCRISPR v2 was a gift from Feng Zhang; Addgene plasmid number 52961; RRID:Addgene_52961). To generate furin knockout (KO) cell lines, lentivirus pseudotyped with vesicular stomatitis virus G protein (VSV-G) were produced by standard triple plasmid transfection using psPax2 and LentiCrisprV2 packaging cassette to package guides against *furin* or scramble control. Following 48 h post-transfection, lentiviral supernatant was filtered and used to transduce HuH7 and HEK 293 cells. The cell lines were transduced for 24 h followed by antibiotic selection with 3 μg/mL puromycin, and a clonal KO population was isolated by serial dilution. *Furin KO* was confirmed by fluorescence microscopy and Western blotting. PCR amplification and sanger sequencing by Eton Bioscience was used to confirm successful mutation within the guide target region (forward primer, 5′-GGGCATCCATCTGTCCATCTATG-3′; and reverse primer, 5′-GAATGGAGACCACAATGCCGTG-3′).

### Western blotting and protein overexpression assay.

To measure the protein expression levels of furin, AAVR, and GPR108, scramble and furin KO cells were lysed in RIPA buffer (25 mM Tris-HCl, pH 7.5, 150 mM NaCl, 1% NP-40, 1% sodium deoxycholate, 0.1% SDS) supplemented with Halt protease inhibitors (Thermo Fisher Scientific 78429). Whole-cell lysate was sonicated on ice for 10 pulses at 40% duty cycle, and cellular debris was cleared by centrifugation. Protein concentrations were determined by Bradford assay (Pierce 23246) and used to normalize all samples. The samples were then heated to 95°C for 3 min in SDS-PAGE loading buffer, separated by SDS-PAGE, and transferred to polyvinylidene difluoride (PVDF) membrane. The membranes were blocked in 5% milk in Tris buffered saline with 0.1% Tween 20 (TBST) and incubated overnight with primary antibodies for GAPDH (1:1,000; Proteintech 60004), furin (1:750; Abcam 105385), KIAA0319L (1:1,000; Abcam 105385), GPR108 (1:1,000; Proteintech 24009), Lamin B2 nuclear loading control (1:1,000; Thermo Fisher Scientific MA1-06104), GAPDH loading control (Proteintech 60004), or α-tubulin loading control (1:10,000; Proteintech 66031). The membranes were washed with TBST and incubated with secondary antibodies conjugated to horseradish peroxidase for 1 h at room temperature (1:5,000; Southern Biotech; rabbit 4010-05; mouse 1010-05). The membranes were then washed and incubated for 5 min with enhanced chemiluminescence substrate (Genesee Scientific 20-300B) and visualized on a Bio-Rad ChemiDoc imaging system. To assess the overexpression of furin in furin KO cell lines, the cells were seeded at 500,000 cells/well in 6-well plates and allowed to adhere overnight. A cDNA sequence obtained from Origene encoding furin was subcloned into the eukaryotic expression vector pcDNA3.1. The cells were then transfected with empty vector pcDNA3.1 or pcDNA3.1-furin using TransIT LT1 transfection reagent (Mirus MIR 2300) according to the manufacturer’s specifications. The samples were then incubated for 48 h at 37°C, and the Western blots were processed as described above.

### Recombinant virus production, purification, and quantification.

Recombinant AAV1, -2, -4, -5, -9, -rh32.33, and sea lion AAV vectors packaging a single-stranded genome encoding firefly luciferase driven by a chicken β-actin (Y) promoter were generated by triple plasmid transfection described previously. Briefly, HEK 293 cells were seeded around 70% confluence in DMEM with 5% FBS and allowed to adhere overnight. The cells were transfected with adenoviral helper plasmid (pXX680; Aldevron), AAV rep-cap plasmid (pLH1, pLH2, pLH4, pLH5, pLH9, pXRrh32.33, and pLHsealion), and the luciferase transgene plasmid (pTR-CBA-Luc; UNC Vector Core) using polyethylenimine (PolySciences 24765-1). Media and/or cell pellets were collected 6 days post-transfection, and viral vectors were harvested by polyethylene glycol precipitation followed by purification by iodixanol density gradient ultracentrifugation. Viral vectors were further subjected to desalting and buffer exchange (Thermo Fisher Scientific 87770) into PBS supplemented with 0.001% pluronic F68 and 1 mM MgCl_2_. To quantify viral titers, purified viral vectors were treated with 10 mg/mL DNase to degrade unencapsidated DNA and subjected to 5% Tween to release viral genomes from capsids. Vector genomes were then quantified by quantitative PCR using a Lightcycler 480 (Roche Applied Sciences). SYBR green I Master Mix (Roche Applied Sciences 4887352001), and primers specific for the luciferase transgene were used for amplification (IDT; forward, 5′-AAAAGCACTCTGATTGACAAATAC-3′; and reverse, 5′-CCTTCGCTTCAAAAAATGGAAC-3′). Seven 10-fold serial dilutions of the luciferase transgene plasmid DNA (starting dilution of 1e_6_ fg/μL) were used to generate a standard curve as described previously. Briefly, the vector genome copy number was calculated by multiplying the number of single-stranded plasmid copies in 1 fg of plasmid (270 copies/fg for pTR-CBA-Luc) by the DNA value acquired from the standard curve. To calculate the number of vector genomes/cell, the vector genome copy number is divided by the number of cells counted at the time of harvest. The percentage of genomes internalized is then calculated by dividing the number of internalized virions by the number of virions bound to the cell surface.

### Phylogenic tree generation.

The phylogenic tree was generated online (http://www.phylogeny.fr/) utilizing the VP1 sequences of a subset of AAVs as input (47). The statistical robustness of the analysis was estimated by bootstrapping with 1,000 replicates. The distance of each branch is proportional to the amount of inferred evolutionary change, and the distance scale of branch length is noted on the bottom left.

### Luciferase transduction assays.

AAVs packaging a single-stranded CBA-luciferase reporter were made by triple plasmid transfection described above. The assays were normalized to cell number where scramble and furin KO cell lines were initially seeded in 24-well plates at 50,000 (HuH7) or 100,000 (HEK 293) cells/well in DMEM supplemented with 10% FBS and penicillin and streptomycin. The cells were then transduced with various AAV serotypes at the MOI indicated and incubated at 37°C for 48 h. For the furin overexpression assay, the cells were transduced with AAV4 24 h post-transfection and incubated for 48 h. The cells were then lysed in 100 μL of passive lysis buffer (Promega E1941), and transduction was assessed by measuring luciferase transgene expression with luciferase assay reagent (Promega E1483) on a Victor X3 microplate reader (PerkinElmer) per the manufacturer’s specifications.

### *In vitro* proteolytic cleavage assay.

Furin cleavage was assayed by incubating purified recombinant AAV4 or AAV2 capsids (5e9 vg total) in citrate-phosphate buffer with 150 mM NaCl at pH 7.4, 6.0, or 5.5 for 30 min at 37°C. The capsids were then treated with recombinant human furin enzyme (2 U/reaction; NEB P8077) in 30-μL reactions supplemented with in 20 mM HEPES, 0.1% Triton X-100, 1 mM CaCl_2,_ 0.2 mM β-mercaptoethanol. The reaction mixtures were incubated at 25°C for 6 h. A known furin substrate, MBP5-paramyosin-ΔSal (NEB E8052), was used as a positive control for furin enzyme activity. Enzymatic reactions were halted by adding SDS-PAGE loading buffer. Capsid proteins were then separated by SDS-PAGE and stained using Coomassie brilliant blue R-250 (Bio-Rad 1610400) or silver stain (Thermo Fisher Scientific LC6070) per the manufacturer’s specifications. Western blotting was completed as described above, and the blots were probed using anti-A1 (AAV VP1) antibody (1:100; American Research Products 03-61056) or B1 (AAV VP1/2/3) antibody (1:100; American Research Products 03-61058).

### Virus binding and uptake assay.

Assessment of AAV binding and uptake were adopted from previous methods with minor changes (29). HuH7 scramble and furin KO cells were seeded in 6-well plates at 200,000 cells/well and allowed to adhere overnight. The cells were then incubated at 4°C for 30 min to halt metabolic processes. The cells were then transduced with AAV4 or AAV2 at 50,000 vg/cell and incubated at 4°C for 1 h to allow viral binding to the cell surface. For binding samples, the cells were collected and washed three times with ice-cold PBS to remove unbound virions. For uptake, the cells were washed three times with ice-cold PBS to remove unbound virions, and fresh, prewarmed DMEM was added to each well. Uptake samples were then incubated at 37°C for 1 h to allow virus internalization. Uptake samples were treated with 1 mL trypsin to dislodge cells and washed three times with PBS. After the final wash, binding and uptake samples were lysed using SideStep lysis and stabilization buffer (Agilent 400900) per the manufacturer’s specifications. Vector genomes were then quantitated by qPCR and analyzed as described above.

### Cytoplasmic and nuclear fractionation.

HuH7 scramble and furin KO cells were seeded as described above for binding and uptake assays. The cells were then transduced with AAV4 at 50,000 vg/cell and incubated overnight at 37°C. The cells were harvested with trypsin-EDTA, washed with PBS, and immediately processed with the NE-PER nuclear and cytoplasmic extraction kit (Thermo Fisher Scientific 78833) to separate cytoplasmic and nuclear extracts per the manufacturer’s specifications. Successful fractionation was confirmed by Western blotting for anti-GAPDH (cytoplasmic protein) and anti-LaminB2 (nuclear envelope protein). Vector genomes were then quantitated by qPCR and analyzed as described above.

### Quantitation of mRNA from AAV vector genomes.

HuH7 scramble and furin KO cells were seeded in 12-well plates at 100,000 cells/well and allowed to adhere overnight. The cells were transduced with AAV4 or AAV2 packaging a luciferase transgene at 20,000 vg/cell. After 48 h, the cells were harvested, and RNA was harvested using Quick-RNA MiniPrep kit (Genesee Scientific 11-327) per the manufacturer’s specifications. cDNA was generated by reverse transcription (Applied Biosystems 4368814) and was analyzed by qPCR with primers targeting luciferase mRNA were used for amplification (IDT; forward, 5′-AAAAGCACTCTGATTGACAAATAC-3′; and reverse, 5′-CCTTCGCTTCAAAAAATGGAAC-3′) and LAMB1 housekeeping gene (forward, 5′-GTTAACAGTCAGGCGCATGGGCC-3′; and reverse, 5′-CCATCAGGGTCACCTCT GGTTCC-3′). mRNA expression fold change was determined with the ΔΔ*CT* method.

### Microscopy and immunofluorescence.

HuH7 scramble and furin KO cells were seeded at 25,000 cells/well in a Lab-Tek II chamber slide (Thermo Fisher Scientific 154453) and allowed to adhere overnight. The cells were washed with PBS supplemented with 1 mM CaCl_2_ and 1 mM MgCl_2_ and then fixed using 10% formalin for 30 min. The cells were then permeabilized using 0.1% Tween 20 in PBS for 20 min followed by blocking with 5% normal goal serum in PBS with for 30 min. The cells were then incubated with primary antibodies for furin (1:100; Proteintech 18413-1-AP), KIAA0319L (1:100; Abcam 105385), or GPR108 (1:100; Proteintech 24009) for 1 h at room temperature. Following three washes with PBS, the cells were incubated with Alexa Fluor secondary antibodies (1:400; Abcam) for 1 h at room temperature. For fluorescent lectin staining, Carbo Free blocking solution (Vector Labs SP-5040-125) was used with fluorescently labeled Jacalin (10 μg/mL; Vector Labs FL-1151-5), Maackia amurensis lectin II (10 μg/mL GlycoMatrix 21511103-1), or *trans*-Golgi Network marker TGN46 (Abcam ab174280). The cells were washed three times with PBS, and slides were mounted with Prolong Gold Antifade Reagent with 4′,6-diamidino-2-phenylindole (DAPI) (Invitrogen P36930). Immunofluorescence was visualized using 10× or 20× Olympus objectives on an ECHO Revolve fluorescence microscope. The cells were counted (70 cells from two or three different microscopy images) and analyzed for relative fluorescent units using ImageJ.

### Competitive inhibition and desialylation assays.

HuH7 scramble and furin KO cell lines were seeded in 24-well plates at 50,000 cells/well and allowed to adhere overnight. The following day, the cells were incubated at 4°C for 30 min before replacing the medium with serum-free DMEM. Lectins concanavalin A (ConA), Sambucus nigra lectin (SNA), peanut agglutinin (PNA), Jacalin (JAC), M. amurensis lectin II (MAL II), or PBS was added to the cells at 100 μg/mL and incubated for 30 min at 4°C. AAV4 was added at 50,000 vg/cell and further incubated for 1 h at 4°C. The medium was then removed, and cells were washed three times with PBS to remove unbound virions. The cells were then incubated at 37°C for 48 h and processed for transduction as described above for the luciferase transduction assays. For the enzymatic removal for sialic acid, the cells were seeded as described previously and treated with either PBS, 0.05 U/mL of neuraminidase (Sigma, 11585886001), or 0.1 U/mL heparinase (Sigma, H2519) for 3 h at 37°C. The cells were then incubated at 4°C for 30 min before AAV4 was added and further incubated for 1 h at 4°C. The medium was then removed, and cells were washed three times with PBS to remove unbound virions. The cells were then incubated at 37°C for 48 h and processed for transduction as described above.
